# Effect of strong wind on laminas and petioles of *Farfugium japonicum* (L.) Kitam. var. *japonicum* (Asteraceae)

**DOI:** 10.3389/fpls.2023.1182266

**Published:** 2023-06-29

**Authors:** Masayuki Shiba, Tsukumo Mizuno, Tatsuya Fukuda

**Affiliations:** Graduate School of Integrative Science and Engineering, Tokyo City University, Setagata, Tokyo, Japan

**Keywords:** AMeDAS, *Farfugium japonicum* var. *japonicum*, leaf, petiole, wind

## Abstract

*Farfugium japonicum* (L.) Kitam. var. *japonicum* grows mainly in the coastal areas of Japan. Meteorological recording data from natural habitats were used to investigate the factors associated with the laminas and petioles of radical leaves of *F. japonicum* var. *japonicum* to avoid or resist higher wind stress. Our morphological and mechanical results indicated that petiole length and petiole cross-sectional area had a weak correlation with wind speed and breaking strength, and the petiole second area moment of inertia did not differ significantly among populations. However, both lamina area and petiole length per petiole cross-sectional area decreased with increasing wind speed, indicating that *F. japonicum* var. *japonicum* resisted or avoided an increase in wind speed outdoors by reducing the lamina area and petiole length per petiole cross-sectional area without qualitative changes in their petioles. The results of this study indicated that densely distributed recording stations of the Automated Meteorological Data Acquisition System (AMeDAS) by the Japan Meteorological Agency can be used for environmental adaptation studies of plants in the field using nearby plant populations.

## 1 Introduction

The genus *Farfugium* Lindl. is a perennial herbaceous species in the Asteraceae family and consists of two species: *F. hiberniflorum* (Makino) Kitam. and *F. japonicum* (L.) Kitam. (Koyama, 1995). *F. hiberniflorum* is an endemic species of Japan that grows in the moist forests of Yakushima and Tanegashima Islands, subtropical islands off the southern coast of Kyushu and part of Kagoshima Prefecture. *F. japonicum* is distributed in Japan, Taiwan, and eastern China and occurs in the margins of forests, as well as roadsides, meadows, riversides, and coastal areas (Koyama, 1995). The four varieties of *F. japonicum* are: *F. japonicum* var. *japonicum*, *F. japonicum* var. *luchuense* (Masamune) Kitam., *F. japonicum* var. *giganteum* (Siebold et Zucc.) Kitam., and *F. japonicum* var. *formosanum* (Hayata) Kitam. These varieties have been used as antipyretics and ingredients in folk remedies for coughs, wound healing, bronchitis, and diarrhoea (Yun et al., 2015). As indicated by Kim et al. (2008), the *F. japonicum* essential oil exerted anti-inflammatory effects, and *F. japonicum* var. *giganteum* and *F. japonicum* var. *formosanum* have been identified as potential chemical and medicinal ingredients (Hsieh et al., 2012; Devkota et al., 2022). In contrast to these medicinal uses, investigation of *F. japonicum* var. *luchuense* has provided insights into the morphological, anatomical, and genetic mechanisms of local plant adaptation. The narrow leaves of *F. japonicum* var. *luchuense* subjected to flash floods after heavy rain showed mechanical selective pressure from water stream in flooded areas along rivers and have adapted to have a decreased number of leaf cells across the width of the leaf (Usukura et al., 1994). In addition, this species has significant intraspecific variation in leaf form, with a trend toward wider leaf base angles as one moves more distant from the riverside (Usukura et al., 1994). Plant groups, including *F. japonicum* var. *luchuense*, inhabiting riverbanks with these leaf morphological characteristics such as narrow leaves are called rheophytes (van Steenis, 1981), and some studies have reported the convergent evolution of many phylogenetically distinct taxa of ferns and angiosperms (Imaichi and Kato, 1992; Yamada et al., 2011; Ohga et al., 2012; Ueda et al., 2012; Yokoyama et al., 2012; Matsui et al., 2013; Shiba et al., 2021). Thus, varieties of *F. japonicum* have been studied for both pharmaceutical purposes and ecological research.

**Figure 1:**
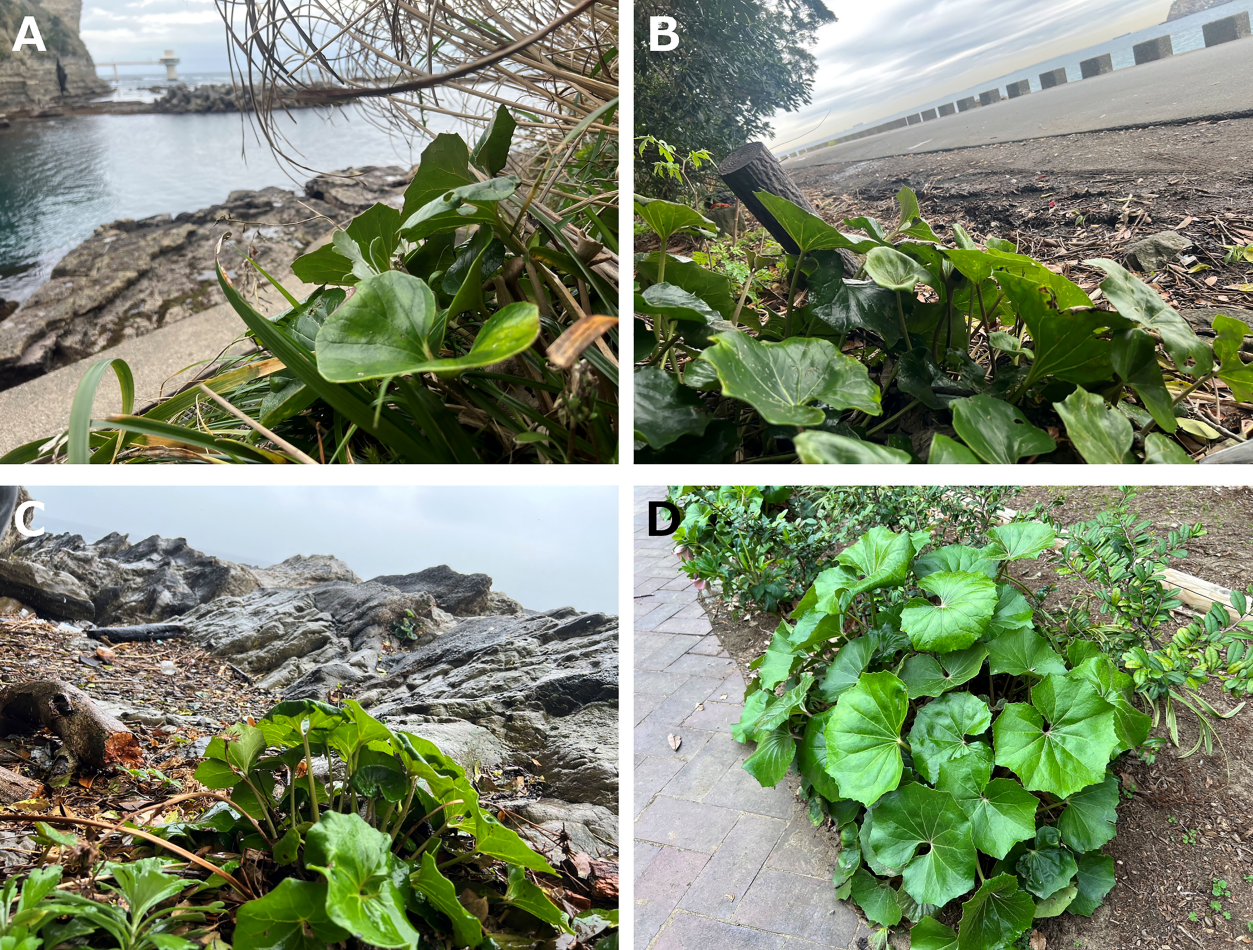
*Farfugium japonicum* (L.) Kitam. var. *japonicum* (Asteraceae). **(A)**: coastal of Ubara; **(B)**: coastal of Kyonan; **(C)**: coastal of Arasaki; **(D)**: inland of Kawasaki.

*F. japonicum* var. *japonicum* grows in the margin of inland forests near the coast, is frequently cultivated for ornamental purposes in gardens (Koyama, 1995), and has hybridised with *F. hiberniflorum* on Yakushima Island (Yamaguchi and Yahara, 1989). *F. japonicum* var. *japonicum* grows in a variety of environments, and it is possible that these ecological transitions from open habitats to coastal conditions have driven morphological and anatomical variation to adapt to different environments (**Figure 1**). In particular, coastal areas are characterised by open, sandy, or rocky habitats with weakly developed soils, which leads to morphological modification. Plant species growing in coastal regions need to adapt to an environment in which drought and wind strongly affect plant growth (Greenway and Munns, 1980). Therefore, it is of interest to clarify the adaptation patterns of *F. japonicum* var. *japonicum* in coastal areas.

Analysing phenotypic plasticity in response to changes in environmental factors is complicated, partially because these conditions may have multiple effects on plants. Sodium (Na^+^) accumulates in the soil of coastal areas, causing osmotic stress between the soil and plant roots, which inhibits water uptake by coastal plants. Sodium (Na^+^) absorbed by the plant body is concentrated in the leaves, reducing the cell’s osmotic potential and causing dehydration or drought stress (Kaspari et al., 2009). Some studies on drought stress in coastal areas have been conducted on coastal adaptation based on morphological and anatomical analyses. Tunala Hayakawa et al. (2012) demonstrated that the epidermal cells of the coastal variety *Aster hispidus* Thunb. var. *insularis* (Makino) Okuyama (Asteraceae) are larger in size and less in number compared to *As. hispidus* var. *hispidus*, and they produce succulent leaves to store water. In this species, Kumekawa et al. (2013) and Sunami et al. (2013) reported that leaf hair on the abaxial side of leaves was correlated to the stomatal density of this variety, and the less hair on the leaf, the lower the stomatal density to avoid transpirational water loss. Ohga et al. (2013) suggested that the coastal population of *Adenophora triphylla* (Thunb.) A.DC. var. *japonica* (Regel) H. Hara (Campanulaceae) had evolved relatively thick leaves *via* a heterochronic process to store water. Shiba et al. (2022) pointed out that coastal populations of *Eurya japonica* Thunb. (Ternstroemiaceae) have small stomata and larger adaxial and abaxial epidermal cells to reduce transpiration during gas exchange and retain moisture within the leaves. Similar results regarding the differentiation between inland and coastal populations were obtained from *Ligstrum japonicum* Thunb. (Oleaceae) (Takizawa et al., 2022) and *L. lucidum* Aiton (Oleaceae) (Takizawa et al., 2023). However, coastal areas are subject to drought as well as wind stress; therefore, it is also necessary to consider morphological changes in response to strong wind stress when discussing adaptation to coastal areas.

Wind flow exerts drag forces on plants causing mechanical stress, and plant responses to wind typically include inhibition of stem elongation and increased stem diameter (Jaffe and Forbes, 1993; Telewski and Pruyn, 1998; Coutand et al., 2000; Anten et al., 2010; Bang et al., 2010). However, wind can also induce responses that are different or even opposite to those induced by pure mechanical stress, such as the production of thinner, more elongated stems under wind loading (Henry and Thomas, 2002; Smith and Ennos, 2003). Wind is a particularly complex environmental factor that has several effects on plants (Ennos, 1997). Wind speeds are stronger in coastal areas than inland areas, and wind-induced stress plays an important role in the speciation of plants adapted to coastal areas (Shiba et al., 2022; Takizawa et al., 2022; Takizawa et al., 2023). However, limited studies have been conducted on plant adaptation to wind-induced mechanical stress in coastal areas. Meguro and Miyawaki (1994) reported that the strain energy per unit volume of branches increased in individuals grown under strong winds compared to those grown under weak winds using the coastal tree species *Celtis sinensis* Pers. (Cannabaceae), *Ilex integra* Thunb. (Aquifoliaceae), *E. japonica*, *Pittosporum tobira* (Thunb.) W.T.Aiton (Pittosporaceae), *Euonymus japonicus* Thunb. (Celastraceae), and *Cinnamomum japonicum* H.Ohba (Lauraceae). However, *F. japonicum* var. *japonicum* may have different responses to the wind because it is a herbaceous species. In general, the most optimised leaf would have a large, flat, and stiff lamina to maximise light capture and a flexible petiole to avoid fractures (Niinemets and Fleck, 2002). The ability to deploy leaves to sunlight regardless of external factors, such as wind, is crucial for plant survival (Roden and Pearcy, 1993; Takenaka, 1994; de Langre, 2008; Vogel, 2009). Niklas (1996) reported that the comparison of sugar maple (*Acer saccharum* Marshall [Aceraceae]) leaves sampled from young trees growing in two locations with different ambient wind speeds showed that individuals in wind-exposed areas had smaller leaf blades and more flexible petioles than those in protected areas. This indicated that wind affected both the leaves and petioles in the sugar maple. Louf et al. (2018) indicated that the ability of the leaf to reduce wind stress at the stem-petiole junction could be achieved by locating the twisting area closer to the lamina; even if the stress is constant throughout the petiole, the strain decreases closer to the stem. This hypothesis is supported by wind-induced bending and twisting stress in red oak (*Quercus rubra* L. [Fagaceae]), American sycamore (*Platanus occidentalis* L. [Platanaceae]), yellow poplar (*Liriodendron tulipifera* L. [Magnoliaceae]), and sugar maple. In addition, some studies have shown that avoidance strategies reduce stem or petiole thickness and flexural rigidity in response to mechanical stress in *Arabidopsis thaliana* (L.) Heynh., *Cecropia schreberiana* Miq. (Urticaceae), *Potentilla reptans* L. (Rosaceae), and *Phaseolus vulgaris* L. (Fabaceae) (Jaffe et al., 1984; Cordero, 1999; Liu et al., 2007; Paul-Victor and Rowe, 2011). Although these studies are important for considering the mechanism by which leaves are not detached from the junction between the lamina and petiole and the stem and petiole, they cannot be directly applied to *F. japonicum* var. *japonicum*; this species has radical leaves that grow perpendicular to the aboveground area from the rhizome, so the static load on the petiole itself may be different from that of plants that extend the petiole horizontally from the stem. In fact, the petioles of *F. japonicum* var. *japonicum* are approximately 30 cm long and carries a leaf which is approximately 10 cm long and 20 cm wide at the tip of the petiole (**Figure 1**). In other plants with radical leaves, comparisons of morphological and mechanical traits under wind stress using *Plantago major* L. (Plantaginaceae) showed that the length of petioles was shorter, but the diameter was not different from that of controls (Anten et al., 2010). This result is important for understanding *F. japonicum* var. *japonicum* adaptations; petioles could also shorten in windy coastal areas and the strength could vary with wind speed.

**Figure 2:**
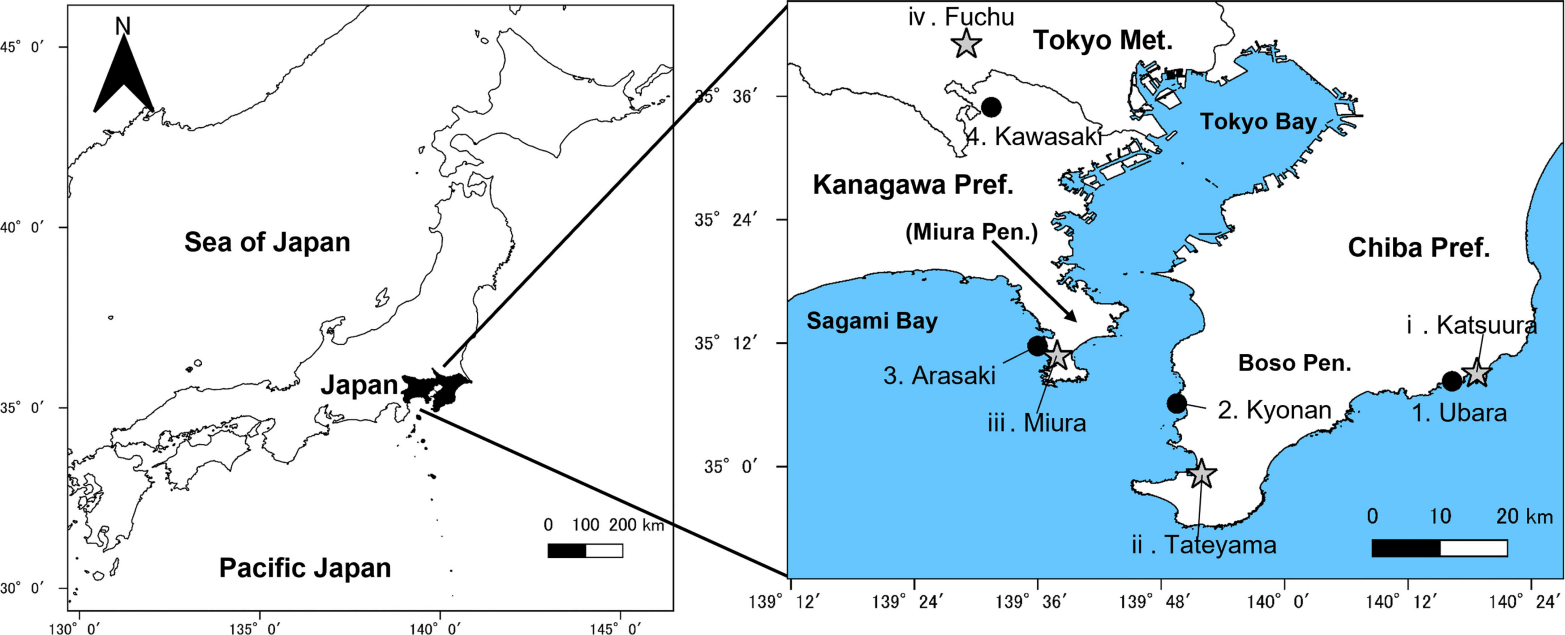
Sampling localities (●) and recording stations of Automated Meteorological Data Acquisition System (AMeDAS) (★). Number corresponds to that given in **Table 1**.

**Figure 3:**
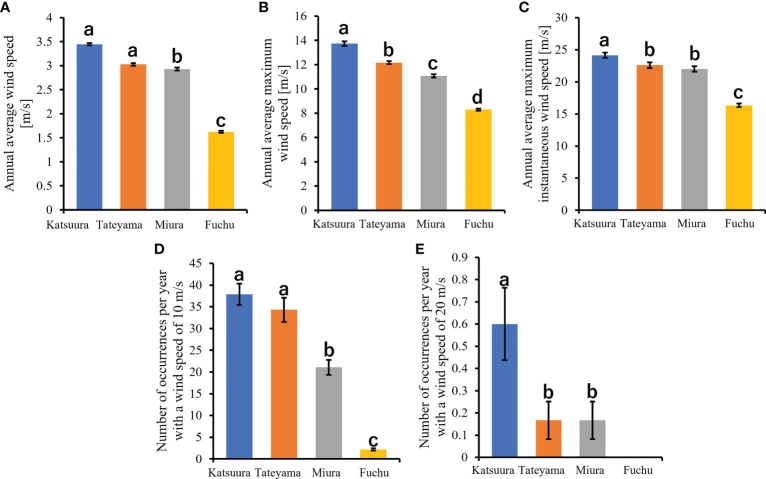
Comparisons of wind speeds of Boso Peninsula and the nearby Miura Peninsula of Kanto District in Japan. **(A)**: annual average wind speed; **(B)**: annual average maximum wind speed; **(C)**: annual average maximum instantaneous wind speed; **(D)**: number of occurrences per year with a wind speed of 10m/s; **(E)**: number of occurrences per year with a wind speed of 20m/s.

Understanding the wind-induced motion of plants is of interest in many fields of plant science and engineering (Roden and Pearcy, 1993; Endalew et al., 2011), and many studies have been conducted in laboratories using wind tunnels (Niinemets and Fleck, 2002; Poulsen and Nordal, 2005; Sack et al., 2008; Jones and Kang, 2015; Ray and Jones, 2018; Wunnenberg et al., 2021). Thorough analysis is key for comparing with the realistic outdoor motion of petioles, as petioles are the most common flexible structure. The Japan Meteorological Agency publishes data and maps of wind speed at the recording stations of the Automated Meteorological Data Acquisition System (AMeDAS). Densely distributed recording stations (1,300 locations) of AMeDAS can approximate field meteorological data without artificial weather manipulation by analysing nearby populations. Because *F. japonicum* var. *japonicum* grows in various places along the coast, the analysis of populations near recording stations of AMeDAS was thought to enable characterisation of differences in laminas and petioles due to wind stress. Using populations as close as possible was preferred because other environmental factors must be considered when the distance between populations is large. Populations of *F. japonicum* var. *japonicum* were found within areas of the east and west sides of Boso Peninsula and the nearby Miura Peninsula of Kanto District in Japan that had different wind speeds, such as average annual wind speed, maximum wind speed, and maximum instantaneous wind speed, as well as the number of occurrences per year with a wind speed of 10 m/s and 20 m/s, according to the AMeDAS data of the Japan Meteorological Agency (**Figures 2**, **3**). The aim of this study was to clarify the effects of wind speed on the laminas and petioles of *F. japonicum* var. *japonicum* in the field based on morphological and mechanical analyses.

## 2 Materials and methods

In this study, wind speed data were obtained from Katsuura, Tateyama, Miura, and Fuchu AMeDAS recording stations near our collection locations (**Figure 2** and **Table 1**). For the analysis of AMeDAS data from the Japan Meteorological Agency, data for the past 30 years in Katsuura, Tateyama, Miura, and Fuchu were used for average annual wind speed, average annual maximum wind speed, and number of occurrences per year with wind speeds of 10 and 20 m/s. Data for the past 10 years were used for the average annual maximum instantaneous wind speed; data from the past 30 years could not be used because there was a difference in the amount of AMeDAS data accumulated among Katsuura, Tateyama, Miura, and Fuchu.

**Table 1:** Sampling and AMeDAS localities used in this study.

Locality name & no.*			Locality	Latitude and longitude	Number of individuals collected
Sampling					
	Ubara	1	Ubara, Katsuura City, Chiba Pref.	35°08’N 140°15’E	32
	Kyonan	2	Kyonan, Awa District, Chiba Pref.	35°06’N 139°49’E	32
	Arasaki	3	Nagai, Yokosuka City, Kanagawa Pref.	35°11’N 139°35’E	33
	Kawasaki	4	Ozenji, Asao-ku, Kawasaki City, Kanagawa Pref.	35°34’N 139°31’E	29
AMeDAS					
	Katsuura	і	Tona, Katsuura City, Chiba Pref.	35°09’N 140°18’E	
	Tateyama	ii	Nagasuka, Tateyama City, Chiba Pref.	34°59’N 139°51’E	
	Miura	iii	Hassemachishimomiyada, Miura City, Kanagawa Pref.	35°10’N 139°37’E	
	Fuchu	iv	Saiwai-cho, Fuchu City, Tokyo Met.	35°41’N 139°45’E	

*: locality number corresponds to that given in **Figure 2**.

**Figure 4:**
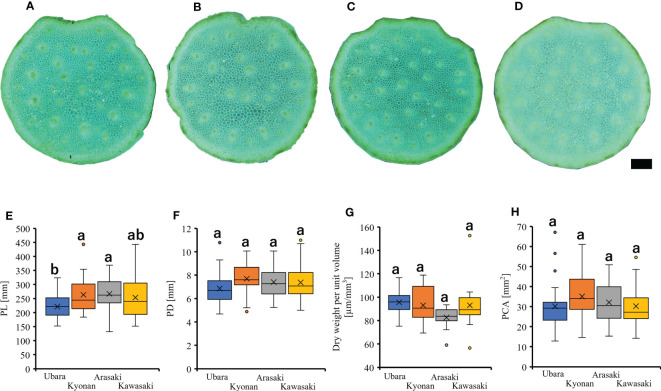
Petiole cross-section of *F. japonicum* var. *japonicum*. **(A)**: Ubara; **(B)**: Kyonan; **(C)**: Arasaki; **(D)**: Kawasaki; Bar = 1 mm. The comparison of petiole morphologies among populations. **(E)**: petiole length; **(F)**: petiole diameter; **(G)**: dry weight per unit volume using mechanical analysis with petiole; **(H)**: petiole cross-sectional area.


Individuals of *F. japonicum* var. *japonicum* examined in this study were collected from the field locations (**Figure 2** and **Table 1**). Leaves were collected one per individual. The inland populations near each coastal area were used as controls. Samples were collected from a total of 126 individuals representing four populations in three coastal areas (Ubara, Kyonan, and Arasaki) and one inland area (Kawasaki) (**Table 1**). For morphological analysis, we measured lamina length, thickness, and area, as well as petiole length, diameter and cross-sectional area for each sample. ImageJ is a widely used application for the analysis of biological images (Bakr, 2005; Igathinathane et al., 2006) and can be used to analyse the area and lamina dimensions. Therefore, we used ImageJ software to measure the lamina length, and area. Leaf thickness was measured using a digimatic outside micrometer (MDC-SB, Mitutoyo, Japan). The petiole cross-section of *F. japonicum* var. *japonicum* at each collection site was sectioned using a razor and observed to be circular, so the elliptical formula was used henceforth (**Figures 4A–D**). Petiole length and diameter were measured using a digimatic caliper (CD-15CXR, Mitutoyo, Japan). The petiole cross-sectional area was calculated according to Eq. 1.

(1) $$\begin{array}{c} PCA=\frac{\pi ab}{4} \end{array}$$

where *a* = petiole long diameter, and *b* = petiole short diameter. After measuring the lamina area, the sample was dried at 100°C for 72 hours in an incubator (FS-405, Advantec, Japan), and the dried weight was measured using an electronic balance (ATX224R, Shimadzu, Japan).

For anatomical analyses, the adaxial surface of the fully expanded lamina was used Suzuki’s Universal Micro-Printing (SUMP) method (Kijima, 1962). Three measurements were taken along the midrib of the lamina, and the average value was calculated. ImageJ software was used to measure the size of epidermal cells.

**Figure 5:**
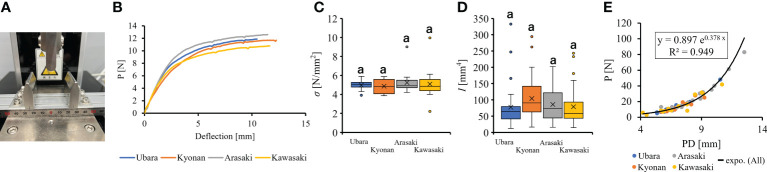
The comparison of petiole mechanical properties among populations. **(A)**: three point bending tester; **(B)**: Representative force-displacement curves of petiole at each location. Measurements approximately 6 mm in petiole diameter are plotted; **(C)**: bending strength; **(D)**: second area moment of inertia; **(E)**: The relationship of petiole diameter and breaking load.


For mechanical analysis, the bending strength of the petiole was measured for each sample using a three-point bending test. The three-point bending test does not require a clamp; instead, the petiole is placed on two supports and a point load is applied to the petiole while the upper support descends at a constant speed to measure displacement and load in bending (**Figure 5A**). A force tester (MCT-1150, A&D, Japan) equipped with a load cell (USM-500N, A&D, Japan) with a resolution of 0.1 N was used in this study. In addition, a three-point bending test was run by attaching an optional jig (JM-B1-500N, A&D, Japan). The span length of the two lower supports was set at 50 cm, so that they were centered in the span relative to the upper supports. Sampling was performed while the upper support was moved downward at a constant speed (10 mm/min). Before conducting the test, *a*, *b* and length of the petiole used for the test were measured as described above and the volume was calculated. The bending strength (σ) was calculated from the results of the three-point bending test using Eq. (2),

(2) $$\begin{array}{c} \sigma =\frac{8PL}{\pi ab^{2}} \end{array}$$

where *P* = breaking load point, and *L* = span (In this experiment, 50 mm fixed). After the test, dry weight was measured on petiole as described above, and dry weight per unit volume was determined. The second area moment of inertia (*I*) was calculated using Eq. 3,


(3) $$\begin{array}{c} I=\frac{\pi ab^{3}}{64} \end{array}$$

Due to sample availability, the number of samples used in the mechanical analysis was performed on 15 individuals at all locations.

## 3 Results

In this study, wind speed data were obtained from Katsuura, Tateyama, Miura, and Fuchu AMeDAS recording stations near our collection locations. For the analysis of AMeDAS data from the Japan Meteorological Agency, data for the past 30 years in Katsuura, Tateyama, Miura, and Fuchu were used for average annual wind speed, average annual maximum wind speed, and number of occurrences per year with wind speeds of 10 and 20 m/s. Data for the past 10 years were used for the average annual maximum instantaneous wind speed; data from the past 30 years could not be used because there was a difference in the amount of AMeDAS data accumulated among Katsuura, Tateyama, Miura, and Fuchu. The results of AMeDAS data analysis showed that average wind speeds from 1991 to 2020 of Katsuura and Tateyama were significantly larger than that of Miura, and all three were significantly larger than Fuchu (**Figure 3A** and **Table 2**). The maximum wind speed from 1991 to 2020 was significantly different in all locations, with the highest in Katsuura, followed by Tateyama, Miura, and Fuchu (**Figure 3B** and **Table 2**). The maximum instantaneous wind speed (2011-2020) was significantly larger in Katsuura than in Tateyama, Miura, and Fuchu (**Figure 3C** and **Table 2**). The number of occurrences per year (1991-2020) with a wind speed of 10 m/s was significantly larger in Katsuura and Tateyama than in Miura and Fuchu (**Figure 3D** and **Table 2**), and wind speed of 20 m/s was significantly larger in Katsuura than in Tateyama, Miura, and Fuchu (**Figure 3E** and **Table 2**).

**Table 2:** Comparison of wind speeds (average ± standard error) of Boso Peninsula and the nearby Miura Peninsula of Kanto District in Japan.

	Katsuura		Tateyama		Miura		Fuchu	
annual average wind speed	3.31 ± 0.02	a	3.03 ± 0.03	a	2.93 ± 0.03	b	1.62 ± 0.02	c
annual average maximum wind speed	13.74 ± 0.19	a	12.16 ± 0.13	b	11.08 ± 0.14	c	7.39 ± 0.11	d
annual average maximum instantaneous wind speed	24.14 ± 0.41	a	22.59 ± 0.44	b	22.00 ± 0.43	b	15.71 ± 0.46	c
Number of occurrences per year with a wind speed of 10 m/s	37.90 ± 2.41	a	34.30 ± 2.78	a	21.07 ± 1.69	b	2.20 ± 0.26	c
Number of occurrences per year with a wind speed of 20 m/s	0.60 ± 1.63	a	0.17 ± 0.08	b	0.17 ± 0.08	b	0	–

Columns marked by different letters differ significantly according to the Holm method (p < 0.05).

**Figure 6:**
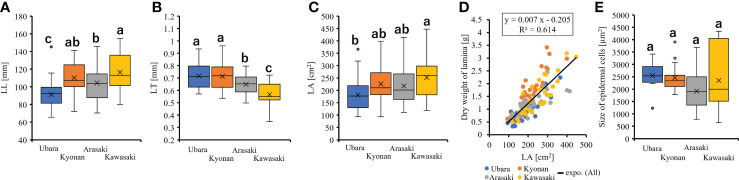
The comparison of lamina morphologies and anatomical among populations. **(A)**: lamina length; **(B)**: lamina thickness; **(C)**: lamina area; **(D)**: the relationship of lamina area and dry weight of lamina; **(E)**: size of epidermal cells.

Comparison of the lamina morphologies of *F. japonicum* var. *japonicum* among the populations showed that lamina length was significantly shorter in the population of Ubara than in those of Kyonan, Arasaki, and Kawasaki (**Figure 6A** and **Table 3**). Lamina thickness in populations of Ubara and Kyonan were significantly larger than those of Arasaki and Kawasaki (**Figure 6B** and **Table 3**). Lamina area was smallest in Ubara, followed by Arasaki, Kyonan, and Kawasaki (**Figure 6C** and **Table 3**). In addition, a strong correlation (R^2 ^= 0.614) was observed between lamina area and dry weight (**Figure 6D**). Our anatomical analyses were indicated that there was no significant difference among Ubara, Kyonan, Arasaki, and Kawasaki (**Figure 6E** and **Table 3**).

**Table 3:** Morphological and anatomical measurements (average ± standard error) of lamina and petiole in *Farfugium japonicum*.

	Ubara		Kyonan		Arasaki		Kawasaki	
lamina								
length [mm]	91.32 ± 2.75	c	110.15 ± 3.04	ab	104.29 ± 3.25	b	116.38 ± 3.82	a
thichness [mm]	0.72 ± 0.02	a	0.71 ± 0.02	a	0.65 ± 0.01	b	0.57 ± 0.02	c
area [mm^2^]	181.33 ± 11.25	b	225.38 ± 12.01	ab	217.33 ± 12.97	ab	251.61 ± 14.95	a
size of epidermal cells [µm^2^]	2549.17 ± 127.43	a	2461.12 ± 144.52	a	1916.99 ± 195.89	a	2347.68 ± 331.39	a
petiole								
length [mm]	221.09 ± 7.38	b	263.22 ± 10.38	a	266.70 ± 10.08	a	253.55 ± 13.03	ab
diameter [mm]	6.86 ± 0.22	a	7.70 ± 0.23	a	7.41 ± 0.22	a	7.37 ± 0.28	a
dry weight per unit volume [µg/mm^3^] (n=15)	95.56 ± 2.73	a	93.03 ± 4.10	a	82.67 ± 2.34	a	93.05 ± 5.22	a
cross-sectional area [mm^2^]	30.15 ± 1.92	a	35.06 ± 1.98	a	32.10 ± 1.80	a	30.21 ± 1.99	a
length/cross-sectional area [mm^-1^]	7.93 ± 0.41	a	8.08 ± 0.44	a	8.85 ± 0.42	a	9.11 ± 0.59	a

Columns marked by different letters differ significantly according to the Holm method (p < 0.05).

Comparison of the petiole morphologies of *F. japonicum* var. *japonicum* among the populations showed that petiole length was significantly longer in populations of Kyonan and Arasaki than in Ubara, while no significant difference was observed with the population of Kawasaki (**Figure 4E** and **Table 3**). No significant differences were observed in the petiole diameter (**Figure 4F** and **Table 3**), dry weight per unit volume using mechanical analyses of petiole (**Figure 4G** and **Table 3**), or petiole cross-sectional area (**Figure 4H** and **Table 3**). The force-displacement curves at the petiole of each location obtained in this study are shown in **Figure 5B**. Comparisons of the petiole mechanical properties of *F. japonicum* var. *japonicum* showed there were no significant differences bending strength (**Figure 5C** and **Table 4**) and second area moment of inertia (**Figure 5D** and **Table 4**). A strong correlation (R^2 ^= 0.949) was observed between petiole diameter and breaking load (**Figure 5E**).

**Table 4:** Mechanical measurements (average ± standard error) of petiole in *Farfugium japonicum*.

	Ubara		Kyonan		Arasaki		Kawasaki	
bending strength [N/mm^2^]	4.97 ± 0.13	a	4.87 ± 0.18	a	5.24 ± 0.30	a	5.09 ± 0.42	a
second area moment of inertia [mm^4^]	77.37 ± 11.39	a	104.07 ± 11.65	a	85.79 ± 9.64	a	78.77 ± 10.95	a

Columns marked by different letters differ significantly according to the Holm method (p < 0.05).

**Figure 7:**
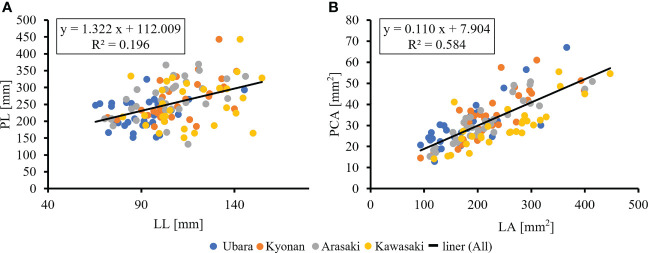
**(A)**: The relationship of lamina length and petiole length. **(B)**: The relationship of lamina area and petiole cross-sectional areas.

**Figure 8:**
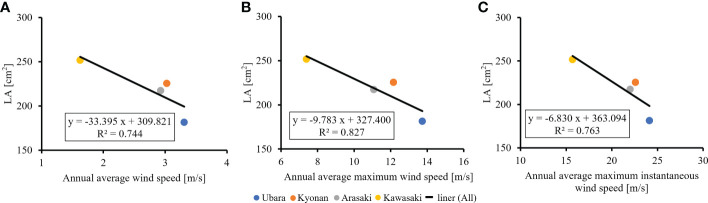
The relationship of lamina area and wind speeds. **(A)**: annual average wind speed; **(B)**: annual average maximum wind speed; **(C)**: annual average maximum instantaneous wind speed.

**Figure 9:**
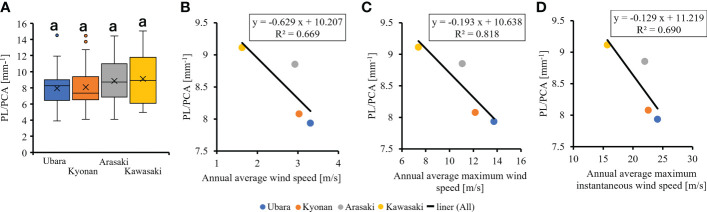
**(A)**: The comparison of petiole length per unit petiole cross-sectional areas among populations. The relationship of petiole length per unit petiole cross-sectional area and wind speeds. **(B)**: annual average wind speed; **(C)**: annual average maximum wind speed; **(D)**: annual average maximum instantaneous wind speed.

The relationships between various morphological traits of *F. japonicum* var. *japonicum* were then examined. Lamina length had a weak correlation (R^2 ^= 0.196) with petiole length (**Figure 7A**), but lamina area had a moderate correlation (R^2 ^= 0.584) with petiole cross-sectional area (**Figure 7B**). Examining the relationship between wind speed and the various morphological traits of *F. japonicum* var. *japonicum* showed that the lamina area was strongly correlated with the average annual wind speed (R^2 ^= 0.744), maximum wind speed (R^2 ^= 0.827), and maximum instantaneous wind speed (R^2 ^= 0.763) (**Figures 8A–C**). However, both petiole length and cross-sectional area had little correlation with any wind speed measurements (**Supplementary Figure 1**). While there was no significant difference in the ratio of petiole length per unit petiole cross-sectional area among populations (**Figure 9A**), there was a strong correlation of the ratio with average annual wind speed (R^2 ^= 0.669), maximum wind speed (R^2 ^= 0.818), and maximum instantaneous wind speed (R^2 ^= 0.690) (**Figures 9B–D**).

## 4 Discussion

Among plant architectures, petioles serve as conduits for the transport of nutrients and sap (Yamada et al., 1999), providing mechanical support for the lamina (Yamada et al., 1999), and adjusting leaf angle or leaf orientation to adapt to variations in the environment (Falster and Westoby, 2003). Petiole lodging of radical leaves can lead to the physical collapse of photosynthetic capacity and can occur spontaneously due to mechanical instability of the leaf structure through external forces such as wind. Wind is one of main environmental factors in petiole lodging. Petiole lodging occurs when a plant is subjected to wind forces greater than the maximum force it can withstand before the petiole breaks, and is the result of wind, lamina area, weight, length, diameter, and mechanical properties of the petiole. Therefore, an understanding all these factors are required to evaluate the petiole lodging resistance of *F. japonicum* var. *japonicum* in different coastal environments. Although our locations were close, the wind speeds in each area, including average annual wind speed, maximum wind speed, maximum instantaneous wind speed, and number of occurrences per year with wind speeds of 10 m/s and 20 m/s, were shown to be different. Our analysis indicated that these wind values were the largest in Katsuura according to the Japan Meteorological Agency records for the past 30 years. Katsuura is located in south-eastern Japan, and low-pressure systems moving from west to east in the Pacific Ocean south of the Japanese archipelago often send southerly to easterly winds directly counter clockwise, especially during typhoons. However, the wind weakens to indirectly reach Tateyama and Miura over the Boso Hills, including the mountains on Mt. Atago, Mt. Kiyosumi-myouken, Mt. Nokogiri, and Mt. Iyogatake on the Boso Peninsula; therefore, there was sufficient difference in the strength of the wind between Katsuura and other locations, even in the coastal area.

It is best to study the effect of plants on the wind using AMeDAS meteorological data at the recording stations, but *F. japonicum* var. *japonicum* could not occur in them. Therefore, we performed the analysis using the closest population from them. AMeDAS meteorological data were commonly used in Japan to assess both the impact of climate change on crop production and crop management practices in the neighboring areas of the recording stations (Seino, 1993; Hasegawa et al., 2011; Kuwagata et al., 2011; Fukuoka and Yoshimoto, 2012; Ohno et al., 2016), suggesting that there is no significant difference in AMeDAS meteorological data used in this study between the neighboring areas and the AMeDAS recording stations. Comparisons were made to clarify the relationship between the wind effects and the lamina area of *F. japonicum* var. *japonicum*. These indicated that the Ubara population near the Katsuura AMeDAS recording station had the smallest lamina area, and there were correlations between wind effects and lamina area. In addition, our results indicated that smaller lamina area was correlated with lighter leaf weight. We found significant differences in lamina area among the populations; lamina area at the lowest wind speed was approximately 1.5 times greater than that at the highest wind speed. Moreover, our results revealed that the lamina area of *F. japonicum* var. *japonicum* showed strong correlations with the average annual wind speed, maximum wind speed, and maximum instantaneous wind speed. Whitehead (1962) showed that the lamina area was greater at the lowest wind speed compared with the highest speed, and our results supported with a small effect at lower wind speeds and much greater effect at higher wind speeds. In addition, our anatomical results indicated that variations in lamina area had not involved changes in cell size because there were no significant differences in cell size among localities. Is it possible for *F. japonicum* var. *japonicum* to vary the lamina area without changing the cell size? Usukura et al. (1994) reported that *F. japonicum* var. *luchuense* had adapted to riverside as a rheophyte by creating narrow lamina without changing cell size, indicating that this report was similar to our results. These indicated that cell size was not involved in the differences in lamina area in *F. japonicum* group, and therefore, we considered that *F. japonicum* var. *japonicum* had adapted to the wind stress by varying the lamina area without changing the cell size.

The petioles of *F. japonicum* var. *japonicum* were related to changes in lamina area between locations. In general, petiole growth is one of the most obvious changes to diminish self-shading at the expense of support; lamina can be sent to a higher position as well as adjusting the angle of the lamina on a branch to avoid overlapping with its neighbours (King and Maindonald, 1999; Bell and Galloway, 2007; Sarlikioti et al., 2011; Zhong et al., 2019). Tsukaya et al. (2002) and Tsukaya (2005) suggested that there is expected to be a positive relationship between petiole and lamina lengths because of a higher proportion of overlap of longer laminas in a given space, which is under genetic control. However, our results indicated that *F. japonicum* var. *japonicum* showed a weak correlation between petiole and lamina length. The pipe-model theory (Shinozaki et al., 1964a; Shinozaki et al., 1964b) indicates that lamina area increases proportionally with increasing petiole cross-sectional area because the large total cross-sectional area of vascular conduits can meet the high demand for photosynthesis or respiration for large laminas (reviewed in Lehnebach et al., 2018). This is supported by several field studies (Li et al., 2008; Ray and Jones, 2018). Our results also revealed a strong correlation between petiole cross-sectional area and lamina area of *F. japonicum* var. *japonicum*. In general, large lamina is not only heavy itself, but also more susceptible to the physical effects of wind, resulting in a greater moment of the upper part of the petiole, which increases the risk of petiole breakage and lodging. In particular, in windy areas, the influence of the wind on the lamina is greater, so the moment of the upper part of the petiole is even greater. Reducing lamina size in strong windy areas is linked to avoid the effects of wind and could adapt without morphological and mechanical changes in petioles, however, smaller lamina reduces the amount of resources obtained through photosynthesis. Conversely, in order to maintain the lamina size in windy areas, it is necessary to increase the strength or the diameter of petioles to withstand the moment at the upper part of them. This is a dilemma between lamina and petiole in windy areas, and our study indicated that *F. japonicum* var. *japonicum* had adapted to windy areas by avoiding the influence of the wind by sacrificing the lamina size without changing the petiole.

Some plant species show resistance to mechanical stress by increasing their stem cross-sectional area and tissue strength (Biddington, 1986; Smith and Ennos, 2003; Anten et al., 2010). This quantifies the distribution of mass in a cross-section with respect to the centre of inertia, which describes the important effect of the size and geometry of the cross-section in mechanics (Mahley et al., 2018). However, our mechanical results for *F. japonicum* var. *japonicum* showed that the bending strength and second moment of inertia were not significantly different among populations, and breaking load increased with thicker petioles. This shows that the petiole becomes thicker by increasing similar supportive tissues such as xylem, sclerenchyma, and collenchyma. These findings implied that the petiole of *F. japonicum* var. *japonicum* would be thicker in the most wind stress locality (Ubara) in our study, but our results did not support this scenario. Some studies have indicated avoidance can occur by a reduction in stem or petiole thickness and flexural rigidity (Jaffe et al., 1984; Cordero, 1999; Liu et al., 2007; Wang et al., 2008; Anten et al., 2010; Paul-Victor and Rowe, 2011). In fact, large plant species, such as trees, are restricted in their ability to bend as they carry heavy loads (Smith and Ennos, 2003; Anten et al., 2009), but small plants are less restricted in this respect and it may be a better strategy to avoid stress (Niklas, 1996; Paul-Victor and Rowe, 2011). *F. japonicum* var. *japonicum* is relatively large herbaceous plant and may be adapted to the highest wind environments through stress-avoidance strategies. Therefore, additional anatomical research on *F. japonicum* var. *japonicum* plays an important role in linking our results with anatomical characteristics.

How plants adapt to fluctuating external forces depends on the strength, frequency, and structure of the plant. Moderate dwarfing and elongation can enhance resistance to wind and rain, and increase lodging resistance in plant species (Jaffe et al., 1984; Cooper and Mendiola, 2004; Liu et al., 2007). Furthermore, plants exposed to wind are suggested to have an adaptive advantage of reducing wind damage due to their lower height compared to plants developed in still air (Russell and Grace, 1978; Telewski and Jaffe, 1986). Zhang et al. (2021) reported changes in the overall morphology of four plant species, *Artemisia ordosica* Krasch (Asteraceae), *Caragana intermedia* Kuang (Fabaceae), *Agriophyllum squarrosum* (L.) Moq. (Chenopodiaceae), and *Salsola ruthenica* Iljin (Chenopodiaceae), among three wind treatments in a field experiment under wind conditions with a monthly average wind speed of approximately 2 m/s and a maximum wind speed of 14.4 m/s in May. In this study, *F. japonicum* var. *japonicum* was shown to adapt to high wind environments by reducing lamina area and the length/cross-sectional area ratio of the leaf stalk as wind speed increased. Three-point bending tests were conducted assuming lodging due to strong winds in this study. However, the petiole affected by wind also experience twisting in addition to bending (Langer et al., 2022). In the future, it will be possible to clarify how petiole of *F. japonicum* var. *japonicum* avoid lodging when subjected to wind loads by evaluating mechanical properties other than bending strength.

*F. japonicum* var. *japonicum* is widely distributed along the coastal areas of Japan (Koyama, 1995), and there are meteorological recording stations for AMeDAS at various locations in these areas. Further studies of *F. japonicum* var. *japonicum* populations at sites with similar wind speeds in Japan are required to clarify morphological changes. This study only partly investigated the strategies used by plants against wind stress by linking the leaf morphology of *F. japonicum* var. *japonicum* and meteorological information in the field using populations near the meteorological recording stations of AMeDAS. Evaluation of morphological traits other than leaves is also important for understanding the environmental adaptations of this plant. *F. japonicum* var. *japonicum* have long scapes, from 30 to 75 cm, that support the inflorescence (Koyama, 1995). Although their radical leaves remain exposed to wind stress for a long period, their scapes appear for a shorter period than the petioles. Investigation into whether differences in the emergence period reflect the differences in adaptive morphology between radical leaves and scapes is required. In addition, Khan et al. (2016) reported that root architecture plays an important role in plant anchorage capability to withstand toppling and overturning by wind loading. Wind could induce thigmomorphogenesis in plants by altering root architecture (Chehab et al., 2009), and the effects of strong winds could increase root biomass allocation (Onoda and Anten, 2011). Whether strong winds also affect the root architecture of *F. japonicum* var. *japonicum* requires further investigation. Addressing these two areas would provide a greater understanding of the morphological and mechanical traits of scapes and roots in *F. japonicum* var. *japonicum* among populations with different wind stresses in the coastal areas.

## Data availability statement

The original contributions presented in the study are included in the article/**Supplementary Material**. Further inquiries can be directed to the corresponding author.

## Author contributions

MS, TM, and TF contributed to conception and design of the study. MS and TM organized the database. MS performed the statistical analysis. TF wrote the first draft of the manuscript. All authors contributed to the article and approved the submitted version.
