# Supplementary material for: Effect of strong wind on laminas and petioles of Farfugium japonicum (L.) Kitam. var. japonicum (Asteraceae)
Source: Front Plant Sci. 2023 Jun 29;14:1182266. doi: 10.3389/fpls.2023.1182266 (PMC10345509; doi:10.3389/fpls.2023.1182266)
Supplement: Supplementary file 1 [file DataSheet_1.docx]

Supplementary Material

Effect of strong wind on laminas and petioles of *Farfugium japonicum* (L.) Kitamura var. *japonicum* (Asteraceae)

# Masayuki Shiba*, Tsukumo Mizuno, Tatsuya Fukuda

# * Correspondence: Masayuki Shiba: msykshiba48@gmail.com

# 1. Supplementary Figure and Tables


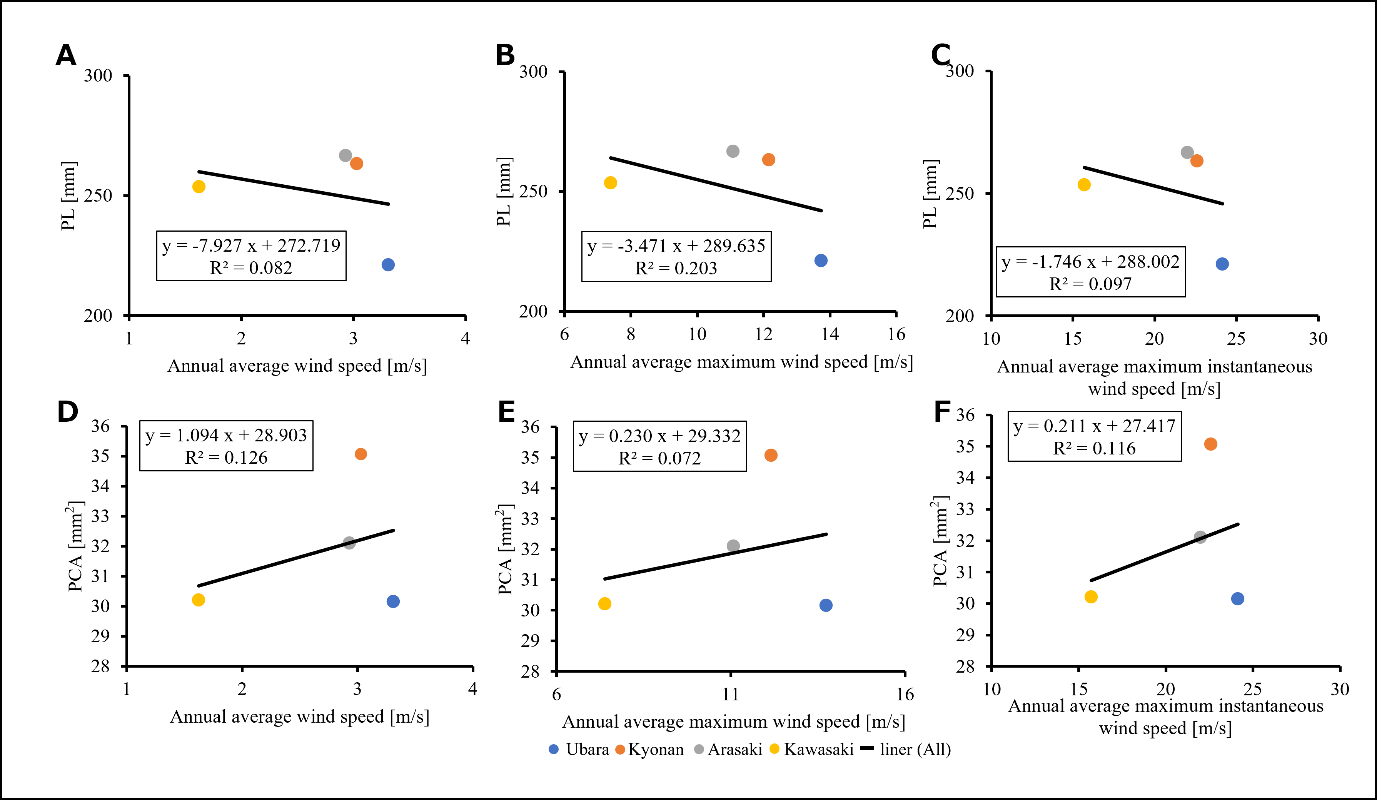
Supplementary Figure 1. The relationship of petiole length, petiole cross-sectional area and wind speeds. (A), (D): annual average wind speed; (B), (E): annual average maximum wind speed; (C), (F): annual average maximum instantaneous wind speed.

Supplementary Table 1. **Annual average wind speed [m/s]**

| **years** | **Katsuura** | **Tateyama** | **Miura** | **Fuchu** |
| --- | --- | --- | --- | --- |
| 1991 | 3.07 | 2.68 | 2.46 | 1.28 |
| 1992 | 3.18 | 2.68 | 2.59 | 1.33 |
| 1993 | 3.15 | 2.75 | 2.67 | 1.42 |
| 1994 | 2.99 | 2.61 | 2.43 | 1.53 |
| 1995 | 3.05 | 2.91 | 2.60 | 1.52 |
| 1996 | 2.95 | 2.84 | 2.44 | 1.65 |
| 1997 | 3.02 | 2.98 | 2.68 | 1.75 |
| 1998 | 3.16 | 2.85 | 2.50 | 1.58 |
| 1999 | 3.19 | 2.91 | 2.49 | 1.69 |
| 2000 | 3.18 | 2.77 | 2.39 | 1.59 |
| 2001 | 3.19 | 2.77 | 2.33 | 1.55 |
| 2002 | 3.30 | 3.08 | 2.84 | 1.61 |
| 2003 | 3.38 | 2.87 | 2.63 | 1.58 |
| 2004 | 3.40 | 3.06 | 2.83 | 1.78 |
| 2005 | 3.13 | 2.93 | 2.68 | 1.59 |
| 2006 | 3.14 | 2.76 | 2.49 | 1.48 |
| 2007 | 3.23 | 2.84 | 2.72 | 1.54 |
| 2008 | 3.18 | 2.70 | 2.51 | 1.63 |
| 2009 | 3.28 | 3.18 | 2.65 | 1.74 |
| 2010 | 3.34 | 3.50 | 3.64 | 1.78 |
| 2011 | 3.36 | 3.39 | 3.59 | 1.76 |
| 2012 | 3.34 | 3.28 | 3.49 | 1.77 |
| 2013 | 3.45 | 3.38 | 3.62 | 1.84 |
| 2014 | 3.75 | 3.39 | 3.58 | 1.71 |
| 2015 | 3.64 | 3.28 | 3.48 | 1.65 |
| 2016 | 3.64 | 3.05 | 3.33 | 1.64 |
| 2017 | 3.69 | 3.25 | 3.43 | 1.63 |
| 2018 | 3.76 | 3.58 | 3.74 | 1.80 |
| 2019 | 3.57 | 3.27 | 3.47 | 1.68 |
| 2020 | 3.56 | 3.30 | 3.56 | 1.63 |

Supplementary Table 2. **Annual average maximum wind speed [m/s]**

| **years** | **Katsuura** | **Tateyama** | **Miura** | **Fuchu** |
| --- | --- | --- | --- | --- |
| 1991 | 12.89 | 11.43 | 10.75 | 6.50 |
| 1992 | 12.53 | 10.77 | 9.67 | 6.33 |
| 1993 | 12.16 | 12.18 | 10.33 | 6.83 |
| 1994 | 11.18 | 10.58 | 9.33 | 7.17 |
| 1995 | 12.46 | 11.47 | 9.75 | 7.08 |
| 1996 | 12.09 | 11.99 | 10.58 | 6.75 |
| 1997 | 11.74 | 11.41 | 10.50 | 8.00 |
| 1998 | 14.36 | 11.84 | 10.25 | 7.00 |
| 1999 | 12.93 | 10.83 | 10.33 | 7.00 |
| 2000 | 11.80 | 11.68 | 10.00 | 6.92 |
| 2001 | 13.09 | 11.32 | 9.50 | 6.17 |
| 2002 | 13.84 | 11.50 | 9.50 | 6.92 |
| 2003 | 13.44 | 11.20 | 10.67 | 6.92 |
| 2004 | 14.27 | 12.53 | 12.25 | 9.17 |
| 2005 | 13.01 | 11.73 | 10.17 | 6.92 |
| 2006 | 12.58 | 11.05 | 9.58 | 6.83 |
| 2007 | 13.13 | 12.08 | 10.83 | 6.92 |
| 2008 | 11.82 | 10.79 | 9.48 | 6.83 |
| 2009 | 13.53 | 13.28 | 10.41 | 7.48 |
| 2010 | 13.23 | 12.52 | 12.33 | 7.88 |
| 2011 | 14.41 | 12.86 | 12.04 | 8.10 |
| 2012 | 15.54 | 12.90 | 13.32 | 8.88 |
| 2013 | 14.45 | 14.33 | 13.59 | 8.76 |
| 2014 | 16.10 | 13.13 | 12.68 | 7.84 |
| 2015 | 15.84 | 12.89 | 11.76 | 7.71 |
| 2016 | 16.51 | 12.93 | 12.78 | 7.60 |
| 2017 | 15.43 | 13.56 | 12.53 | 7.39 |
| 2018 | 15.83 | 13.62 | 12.95 | 9.03 |
| 2019 | 17.24 | 14.36 | 13.24 | 7.57 |
| 2020 | 14.68 | 11.98 | 11.19 | 7.25 |

Supplementary Table 3. **Annual average maximum instantaneous wind speed [m/s]**

| **years** | **Katsuura** | **Tateyama** | **Miura** | **Fuchu** |
| --- | --- | --- | --- | --- |
| 2011 | 23.30 | 21.58 | 20.82 | 15.91 |
| 2012 | 25.03 | 22.51 | 22.72 | 17.53 |
| 2013 | 25.39 | 24.15 | 23.48 | 17.15 |
| 2014 | 24.89 | 22.34 | 21.96 | 14.85 |
| 2015 | 23.66 | 21.62 | 20.18 | 14.10 |
| 2016 | 24.15 | 21.93 | 21.74 | 15.09 |
| 2017 | 24.03 | 23.34 | 22.50 | 14.67 |
| 2018 | 24.90 | 23.64 | 23.33 | 18.34 |
| 2019 | 24.98 | 24.70 | 23.52 | 15.03 |
| 2020 | 21.03 | 20.14 | 19.79 | 14.44 |

Supplementary Table 4. **Number of occurrences per year with a wind speed of 10 m/s**

| **years** | **Katsuura** | **Tateyama** | **Miura** | **Fuchu** |
| --- | --- | --- | --- | --- |
| 1991 | 7.12 | 6.85 | 4.66 | 0.27 |
| 1992 | 8.22 | 4.66 | 3.01 | 0.00 |
| 1993 | 7.12 | 9.59 | 4.66 | 0.27 |
| 1994 | 5.21 | 4.11 | 3.29 | 0.27 |
| 1995 | 6.03 | 5.48 | 2.74 | 0.27 |
| 1996 | 6.85 | 8.22 | 4.38 | 0 |
| 1997 | 4.93 | 7.40 | 5.48 | 0.82 |
| 1998 | 8.22 | 4.93 | 4.66 | 0.82 |
| 1999 | 6.58 | 4.38 | 3.56 | 0.27 |
| 2000 | 6.30 | 5.21 | 3.29 | 0.00 |
| 2001 | 8.77 | 5.21 | 1.92 | 0.00 |
| 2002 | 8.49 | 8.77 | 3.84 | 0.27 |
| 2003 | 10.96 | 7.67 | 4.66 | 0.27 |
| 2004 | 12.88 | 8.49 | 9.32 | 2.47 |
| 2005 | 9.59 | 5.75 | 4.38 | 0.27 |
| 2006 | 10.14 | 7.12 | 3.56 | 0.27 |
| 2007 | 10.96 | 6.30 | 5.21 | 0.27 |
| 2008 | 5.75 | 3.84 | 3.56 | 0 |
| 2009 | 10.96 | 13.15 | 4.11 | 0.55 |
| 2010 | 14.52 | 13.70 | 9.32 | 0.82 |
| 2011 | 12.88 | 13.15 | 8.22 | 0.55 |
| 2012 | 10.68 | 13.70 | 7.12 | 1.37 |
| 2013 | 16.16 | 15.89 | 10.96 | 1.37 |
| 2014 | 16.99 | 15.62 | 10.41 | 0.82 |
| 2015 | 15.62 | 11.51 | 6.03 | 0 |
| 2016 | 13.42 | 12.05 | 7.67 | 0.55 |
| 2017 | 12.33 | 13.70 | 7.40 | 0.27 |
| 2018 | 17.53 | 14.52 | 8.49 | 1.10 |
| 2019 | 13.15 | 13.97 | 7.95 | 0 |
| 2020 | 13.15 | 16.99 | 9.32 | 0 |

Supplementary Table 5. **Number of occurrences per year with a wind speed of 20 m/s**

| **years** | **Katsuura** | **Tateyama** | **Miura** | **Fuchu** |
| --- | --- | --- | --- | --- |
| 1991 | 0 | 0 | 0 | 0 |
| 1992 | 0 | 0 | 0 | 0 |
| 1993 | 0 | 0 | 0 | 0 |
| 1994 | 0 | 0 | 0 | 0 |
| 1995 | 0 | 0 | 0 | 0 |
| 1996 | 0 | 0.27 | 0 | 0 |
| 1997 | 0 | 0 | 0 | 0 |
| 1998 | 0.27 | 0 | 0 | 0 |
| 1999 | 0 | 0 | 0 | 0 |
| 2000 | 0 | 0 | 0 | 0 |
| 2001 | 0 | 0 | 0 | 0 |
| 2002 | 0.27 | 0 | 0 | 0 |
| 2003 | 0 | 0 | 0 | 0 |
| 2004 | 0.27 | 0 | 0 | 0 |
| 2005 | 0.27 | 0 | 0 | 0 |
| 2006 | 0 | 0 | 0 | 0 |
| 2007 | 0 | 0 | 0 | 0 |
| 2008 | 0 | 0 | 0 | 0 |
| 2009 | 0 | 0 | 0 | 0 |
| 2010 | 0 | 0 | 0 | 0 |
| 2011 | 0.27 | 0 | 0 | 0 |
| 2012 | 0 | 0 | 0 | 0 |
| 2013 | 0.27 | 0.27 | 0.27 | 0 |
| 2014 | 0.27 | 0.27 | 0.27 | 0 |
| 2015 | 0.27 | 0 | 0 | 0 |
| 2016 | 0.55 | 0 | 0 | 0 |
| 2017 | 1.10 | 0 | 0 | 0 |
| 2018 | 0.27 | 0 | 0.27 | 0 |
| 2019 | 0.55 | 0.55 | 0.55 | 0 |
| 2020 | 0.27 | 0 | 0 | 0 |

Supplementary Table 6. **Lamina length [mm]**

| **Sample** | **Ubara** | **Kyonan** | **Arasaki** | **Kawasaki** |
| --- | --- | --- | --- | --- |
| 1 | 97.04 | 126.62 | 107.28 | 114.35 |
| 2 | 94.27 | 100.22 | 110.23 | 106.75 |
| 3 | 115.48 | 139.91 | 120.67 | 102.28 |
| 4 | 90.77 | 98.62 | 106.07 | 129.10 |
| 5 | 81.11 | 100.10 | 100.60 | 84.39 |
| 6 | 99.56 | 93.54 | 103.13 | 142.95 |
| 7 | 98.07 | 113.78 | 115.09 | 80.07 |
| 8 | 92.89 | 120.14 | 70.39 | 103.68 |
| 9 | 99.91 | 96.78 | 135.83 | 102.89 |
| 10 | 96.86 | 72.05 | 121.12 | 140.82 |
| 11 | 86.51 | 101.70 | 145.81 | 135.68 |
| 12 | 85.58 | 100.54 | 113.34 | 154.76 |
| 13 | 77.58 | 100.32 | 122.79 | 141.41 |
| 14 | 100.33 | 126.27 | 124.65 | 123.52 |
| 15 | 91.45 | 134.27 | 97.75 | 101.48 |
| 16 | 96.19 | 119.54 | 107.02 | 105.33 |
| 17 | 85.71 | 101.28 | 103.04 | 113.08 |
| 18 | 106.08 | 97.06 | 108.37 | 101.22 |
| 19 | 99.77 | 131.70 | 113.98 | 135.83 |
| 20 | 82.06 | 89.99 | 136.33 | 112.97 |
| 21 | 84.74 | 120.60 | 103.18 | 149.94 |
| 22 | 98.89 | 141.13 | 84.76 | 118.53 |
| 23 | 145.25 | 104.75 | 102.94 | 99.66 |
| 24 | 109.93 | 133.13 | 87.80 | 101.38 |
| 25 | 72.38 | 107.74 | 114.11 | 112.13 |
| 26 | 92.97 | 108.80 | 81.91 | 144.62 |
| 27 | 82.81 | 77.11 | 73.91 | 131.98 |
| 28 | 74.19 | 133.08 | 102.11 | 91.78 |
| 29 | 74.04 | 102.50 | 87.34 | 92.41 |
| 30 | 65.42 | 116.00 | 103.69 | － |
| 31 | 67.76 | 106.28 | 74.00 | － |
| 32 | 76.80 | 109.38 | 74.91 | － |
| 33 | － | － | 87.27 | － |

Supplementary Table 7. **Lamina thickness [mm]**

| **Sample** | **Ubara** | **Kyonan** | **Arasaki** | **Kawasaki** |
| --- | --- | --- | --- | --- |
| 1 | 0.63 | 0.54 | 0.70 | 0.53 |
| 2 | 0.63 | 0.85 | 0.68 | 0.44 |
| 3 | 0.67 | 0.75 | 0.78 | 0.72 |
| 4 | 0.57 | 0.63 | 0.63 | 0.61 |
| 5 | 0.60 | 0.72 | 0.64 | 0.38 |
| 6 | 0.58 | 0.60 | 0.80 | 0.65 |
| 7 | 0.77 | 0.65 | 0.71 | 0.35 |
| 8 | 0.74 | 0.85 | 0.59 | 0.40 |
| 9 | 0.60 | 0.62 | 0.50 | 0.58 |
| 10 | 0.71 | 0.64 | 0.50 | 0.66 |
| 11 | 0.65 | 0.60 | 0.65 | 0.61 |
| 12 | 0.80 | 0.84 | 0.65 | 0.59 |
| 13 | 0.59 | 0.61 | 0.68 | 0.72 |
| 14 | 0.63 | 0.81 | 0.71 | 0.60 |
| 15 | 0.66 | 0.96 | 0.65 | 0.55 |
| 16 | 0.83 | 0.80 | 0.67 | 0.54 |
| 17 | 0.93 | 0.66 | 0.74 | 0.54 |
| 18 | 0.80 | 0.71 | 0.73 | 0.59 |
| 19 | 0.61 | 0.67 | 0.64 | 0.65 |
| 20 | 0.57 | 0.73 | 0.67 | 0.54 |
| 21 | 0.70 | 0.78 | 0.57 | 0.69 |
| 22 | 0.74 | 0.77 | 0.69 | 0.66 |
| 23 | 0.77 | 0.79 | 0.60 | 0.68 |
| 24 | 0.80 | 0.62 | 0.54 | 0.52 |
| 25 | 0.91 | 0.80 | 0.69 | 0.52 |
| 26 | 0.71 | 0.72 | 0.59 | 0.54 |
| 27 | 0.75 | 0.62 | 0.75 | 0.52 |
| 28 | 0.75 | 0.74 | 0.55 | 0.49 |
| 29 | 0.84 | 0.72 | 0.57 | 0.54 |
| 30 | 0.65 | 0.68 | 0.60 | － |
| 31 | 0.80 | 0.77 | 0.53 | － |
| 32 | 0.93 | 0.62 | 0.71 | － |
| 33 | － | － | 0.72 | － |

Supplementary Table 8. **Lamina area [mm^2^]**

| **Sample** | **Ubara** | **Kyonan** | **Arasaki** | **Kawasaki** |
| --- | --- | --- | --- | --- |
| 1 | 184.91 | 182.60 | 297.52 | 287.39 |
| 2 | 236.92 | 187.08 | 224.42 | 316.94 |
| 3 | 317.92 | 271.83 | 298.57 | 219.16 |
| 4 | 168.73 | 163.31 | 230.05 | 256.11 |
| 5 | 119.72 | 173.05 | 211.99 | 169.72 |
| 6 | 199.79 | 170.60 | 265.39 | 353.90 |
| 7 | 191.73 | 212.38 | 214.21 | 144.19 |
| 8 | 216.18 | 269.23 | 120.53 | 272.11 |
| 9 | 227.43 | 199.54 | 399.80 | 221.04 |
| 10 | 169.32 | 93.24 | 414.14 | 399.70 |
| 11 | 132.08 | 182.22 | 315.41 | 291.92 |
| 12 | 137.10 | 208.64 | 255.73 | 351.42 |
| 13 | 126.66 | 174.39 | 267.43 | 447.26 |
| 14 | 219.82 | 392.73 | 298.38 | 282.67 |
| 15 | 173.15 | 296.91 | 174.29 | 189.29 |
| 16 | 248.75 | 300.54 | 186.16 | 156.09 |
| 17 | 129.93 | 165.80 | 180.06 | 206.70 |
| 18 | 218.99 | 202.66 | 189.28 | 206.02 |
| 19 | 187.11 | 293.86 | 205.79 | 328.00 |
| 20 | 156.00 | 183.82 | 284.94 | 282.71 |
| 21 | 180.73 | 227.51 | 197.76 | 284.65 |
| 22 | 196.90 | 310.07 | 177.94 | 261.46 |
| 23 | 365.96 | 276.69 | 186.55 | 172.91 |
| 24 | 290.75 | 243.79 | 153.95 | 184.23 |
| 25 | 108.48 | 215.26 | 229.64 | 179.12 |
| 26 | 192.52 | 193.62 | 154.27 | 304.39 |
| 27 | 115.25 | 116.60 | 117.63 | 259.55 |
| 28 | 129.74 | 398.84 | 164.89 | 149.79 |
| 29 | 141.88 | 204.03 | 161.87 | 118.37 |
| 30 | 93.19 | 227.24 | 201.07 | － |
| 31 | 113.36 | 238.77 | 121.43 | － |
| 32 | 111.41 | 235.25 | 110.93 | － |
| 33 | － | － | 159.74 | － |

Supplementary Table 9. **Size of epidermal cells [µm^2^]**

| **Sample** | **Ubara** | **Kyonan** | **Arasaki** | **Kawasaki** |
| --- | --- | --- | --- | --- |
| 1 | 2686.973 | 2384.229 | 772.4129 | 1624.357 |
| 2 | 2945.678 | 2245.787 | 1100.859 | 1014.94 |
| 3 | 3418.385 | 2110.932 | 2011.222 | 4321.163 |
| 4 | 2741.435 | 2468.506 | 1726.145 | 4321.163 |
| 5 | 2418.55 | 2482.316 | 1352.564 | 1130.369 |
| 6 | 2247.01 | 3083.444 | 2116.48 | 4338.171 |
| 7 | 2401.311 | 3906.575 | 888.1747 | 1581.066 |
| 8 | 1230.232 | 3251.384 | 2498.678 | 1909.109 |
| 9 | 2536.009 | 2127.925 | 2539.994 | 4047.535 |
| 10 | 2912.045 | 2560.158 | 1720.544 | 2144.127 |
| 11 | 3047.544 | 2338.659 | 1772.661 | 1513.74 |
| 12 | 2275.145 | 1951.695 | 2772.719 | 2533.362 |
| 13 | 2640.869 | 2061.141 | 1909.354 | 2093.637 |
| 14 | 2513.229 | 1777.739 | 1895.148 | 1998.293 |
| 15 | 2223.183 | 2.17E+03 | 3.68E+03 | 6.44E+02 |

Supplementary Table 10. **Petiole length [mm]**

| **Sample** | **Ubara** | **Kyonan** | **Arasaki** | **Kawasaki** |
| --- | --- | --- | --- | --- |
| 1 | 265 | 282 | 336 | 312 |
| 2 | 257 | 241 | 236 | 329 |
| 3 | 265 | 237 | 369 | 199 |
| 4 | 188 | 225 | 236 | 187 |
| 5 | 187 | 321 | 262 | 334 |
| 6 | 244 | 218 | 295 | 443 |
| 7 | 153 | 266 | 132 | 213 |
| 8 | 189 | 309 | 207 | 319 |
| 9 | 158 | 230 | 326 | 215 |
| 10 | 218 | 211 | 351 | 224 |
| 11 | 207 | 245 | 333 | 240 |
| 12 | 152 | 228 | 335 | 328 |
| 13 | 195 | 212 | 297 | 347 |
| 14 | 223 | 274 | 251 | 295 |
| 15 | 201 | 302 | 284 | 214 |
| 16 | 197 | 185 | 298 | 232 |
| 17 | 163 | 271 | 252 | 177 |
| 18 | 233 | 184 | 304 | 292 |
| 19 | 226 | 443 | 255 | 296 |
| 20 | 255 | 233 | 267 | 151 |
| 21 | 253 | 354 | 367 | 165 |
| 22 | 251 | 348 | 237 | 165 |
| 23 | 293 | 288 | 233 | 164 |
| 24 | 220 | 294 | 294 | 252 |
| 25 | 166 | 301 | 316 | 288 |
| 26 | 324 | 332 | 265 | 269 |
| 27 | 197 | 204 | 206 | 298 |
| 28 | 254 | 326 | 250 | 217 |
| 29 | 244 | 199 | 244 | 188 |
| 30 | 247 | 205 | 167 | － |
| 31 | 252 | 212 | 208 | － |
| 32 | 198 | 243 | 186 | － |
| 33 | － | － | 202 | － |

Supplementary Table 11. **Petiole diameter [mm]**

| **Sample** | **Ubara** | **Kyonan** | **Arasaki** | **Kawasaki** |
| --- | --- | --- | --- | --- |
| 1 | 7.40 | 7.74 | 9.27 | 7.73 |
| 2 | 7.63 | 7.93 | 7.78 | 7.74 |
| 3 | 7.34 | 8.19 | 9.55 | 7.31 |
| 4 | 5.28 | 5.82 | 6.84 | 6.38 |
| 5 | 4.69 | 6.19 | 7.41 | 6.22 |
| 6 | 8.02 | 6.00 | 8.14 | 10.69 |
| 7 | 6.73 | 7.53 | 7.28 | 5.72 |
| 8 | 7.11 | 8.78 | 6.01 | 7.29 |
| 9 | 6.68 | 6.29 | 10.07 | 6.60 |
| 10 | 6.20 | 4.88 | 9.76 | 8.57 |
| 11 | 5.19 | 7.59 | 9.05 | 7.42 |
| 12 | 6.52 | 7.16 | 7.76 | 10.99 |
| 13 | 5.74 | 7.39 | 8.55 | 9.66 |
| 14 | 7.34 | 9.78 | 8.29 | 8.44 |
| 15 | 6.47 | 9.52 | 7.19 | 7.07 |
| 16 | 8.96 | 8.80 | 7.42 | 9.51 |
| 17 | 6.30 | 8.01 | 6.81 | 6.08 |
| 18 | 7.64 | 6.66 | 7.26 | 6.61 |
| 19 | 7.12 | 8.97 | 8.04 | 8.23 |
| 20 | 6.39 | 7.49 | 8.61 | 6.43 |
| 21 | 6.72 | 8.16 | 6.25 | 8.23 |
| 22 | 8.10 | 10.06 | 7.22 | 6.76 |
| 23 | 10.78 | 8.38 | 7.74 | 6.90 |
| 24 | 9.31 | 9.57 | 6.64 | 5.72 |
| 25 | 5.65 | 7.39 | 7.35 | 6.67 |
| 26 | 7.56 | 7.55 | 6.30 | 8.10 |
| 27 | 5.74 | 5.21 | 5.61 | 6.84 |
| 28 | 6.71 | 9.39 | 6.09 | 5.02 |
| 29 | 6.56 | 7.61 | 6.30 | 4.99 |
| 30 | 5.45 | 7.19 | 6.48 | － |
| 31 | 6.44 | 7.26 | 5.61 | － |
| 32 | 5.85 | 7.96 | 5.25 | － |
| 33 | － | － | 6.66 | － |

Supplementary Table 12. **Dry weight per unit volume of petiole [µg/mm^3^]**

| **Sample** | **Ubara** | **Kyonan** | **Arasaki** | **Kawasaki** |
| --- | --- | --- | --- | --- |
| 1 | 100.81 | 69.55 | 58.97 | 85.91 |
| 2 | 101.35 | 69.30 | 74.56 | 86.32 |
| 3 | 108.96 | 76.95 | 83.61 | 101.63 |
| 4 | 89.47 | 113.39 | 93.50 | 98.45 |
| 5 | 94.52 | 104.94 | 87.99 | 99.66 |
| 6 | 116.69 | 110.15 | 89.23 | 152.65 |
| 7 | 75.20 | 83.61 | 93.17 | 94.47 |
| 8 | 81.05 | 86.21 | 82.47 | 88.15 |
| 9 | 83.72 | 90.60 | 83.71 | 56.48 |
| 10 | 92.35 | 83.88 | 81.59 | 89.26 |
| 11 | 94.39 | 82.85 | 79.83 | 84.93 |
| 12 | 100.42 | 97.75 | 87.27 | 104.38 |
| 13 | 96.44 | 118.88 | 72.12 | 76.54 |
| 14 | 95.86 | 98.23 | 80.82 | 83.86 |
| 15 | 102.19 | 109.15 | 91.18 | 93.05 |

Supplementary Table 13. **Petiole cross-sectional area [mm^2^]**

| **Sample** | **Ubara** | **Kyonan** | **Arasaki** | **Kawasaki** |
| --- | --- | --- | --- | --- |
| 1 | 31.56 | 33.49 | 49.50 | 34.68 |
| 2 | 32.15 | 36.88 | 30.53 | 32.48 |
| 3 | 30.13 | 31.60 | 50.70 | 29.51 |
| 4 | 20.49 | 18.63 | 26.75 | 37.68 |
| 5 | 12.90 | 23.41 | 28.47 | 23.78 |
| 6 | 36.26 | 20.98 | 46.04 | 48.57 |
| 7 | 32.02 | 33.23 | 32.17 | 15.40 |
| 8 | 31.32 | 48.21 | 19.58 | 27.12 |
| 9 | 24.63 | 22.89 | 47.26 | 22.10 |
| 10 | 26.56 | 14.59 | 50.90 | 45.01 |
| 11 | 18.85 | 31.95 | 39.36 | 32.15 |
| 12 | 29.92 | 27.59 | 35.09 | 55.42 |
| 13 | 18.66 | 31.32 | 49.02 | 54.56 |
| 14 | 31.41 | 51.19 | 40.47 | 31.07 |
| 15 | 22.76 | 46.57 | 31.43 | 24.88 |
| 16 | 47.80 | 45.18 | 25.07 | 41.11 |
| 17 | 22.35 | 37.23 | 28.95 | 24.23 |
| 18 | 32.05 | 28.21 | 29.01 | 24.79 |
| 19 | 34.50 | 42.02 | 37.10 | 34.06 |
| 20 | 24.60 | 31.45 | 41.72 | 26.42 |
| 21 | 28.58 | 40.53 | 25.43 | 32.07 |
| 22 | 39.55 | 61.03 | 33.37 | 26.92 |
| 23 | 67.04 | 44.22 | 33.98 | 27.20 |
| 24 | 56.53 | 57.55 | 26.77 | 16.75 |
| 25 | 24.21 | 34.57 | 32.88 | 21.18 |
| 26 | 35.88 | 34.57 | 21.41 | 30.18 |
| 27 | 23.74 | 16.03 | 18.30 | 26.61 |
| 28 | 30.28 | 46.61 | 22.72 | 15.83 |
| 29 | 27.71 | 35.14 | 21.93 | 14.22 |
| 30 | 20.72 | 30.04 | 27.66 | － |
| 31 | 26.65 | 30.34 | 17.09 | － |
| 32 | 23.03 | 34.83 | 15.29 | － |
| 33 | － | － | 23.36 | － |

Supplementary Table 14. **Bending strength [N/mm^2^]**

| **Sample** | **Ubara** | **Kyonan** | **Arasaki** | **Kawasaki** |
| --- | --- | --- | --- | --- |
| 1 | 100.81 | 69.55 | 58.97 | 85.91 |
| 2 | 101.35 | 69.30 | 74.56 | 86.32 |
| 3 | 108.96 | 76.95 | 83.61 | 101.63 |
| 4 | 89.47 | 113.39 | 93.50 | 98.45 |
| 5 | 94.52 | 104.94 | 87.99 | 99.66 |
| 6 | 116.69 | 110.15 | 89.23 | 152.65 |
| 7 | 75.20 | 83.61 | 93.17 | 94.47 |
| 8 | 81.05 | 86.21 | 82.47 | 88.15 |
| 9 | 83.72 | 90.60 | 83.71 | 56.48 |
| 10 | 92.35 | 83.88 | 81.59 | 89.26 |
| 11 | 94.39 | 82.85 | 79.83 | 84.93 |
| 12 | 100.42 | 97.75 | 87.27 | 104.38 |
| 13 | 96.44 | 118.88 | 72.12 | 76.54 |
| 14 | 95.86 | 98.23 | 80.82 | 83.86 |
| 15 | 102.19 | 109.15 | 91.18 | 93.05 |

Supplementary Table 15. **Second area moment of inertia [mm^4^]**

| **Sample** | **Ubara** | **Kyonan** | **Arasaki** | **Kawasaki** |
| --- | --- | --- | --- | --- |
| 1 | 100.81 | 69.55 | 58.97 | 85.91 |
| 2 | 101.35 | 69.30 | 74.56 | 86.32 |
| 3 | 108.96 | 76.95 | 83.61 | 101.63 |
| 4 | 89.47 | 113.39 | 93.50 | 98.45 |
| 5 | 94.52 | 104.94 | 87.99 | 99.66 |
| 6 | 116.69 | 110.15 | 89.23 | 152.65 |
| 7 | 75.20 | 83.61 | 93.17 | 94.47 |
| 8 | 81.05 | 86.21 | 82.47 | 88.15 |
| 9 | 83.72 | 90.60 | 83.71 | 56.48 |
| 10 | 92.35 | 83.88 | 81.59 | 89.26 |
| 11 | 94.39 | 82.85 | 79.83 | 84.93 |
| 12 | 100.42 | 97.75 | 87.27 | 104.38 |
| 13 | 96.44 | 118.88 | 72.12 | 76.54 |
| 14 | 95.86 | 98.23 | 80.82 | 83.86 |
| 15 | 102.19 | 109.15 | 91.18 | 93.05 |

Supplementary Table 16. **PL/PCA [mm^-1^]**

| **Sample** | **Ubara** | **Kyonan** | **Arasaki** | **Kawasaki** |
| --- | --- | --- | --- | --- |
| 1 | 8.40 | 8.42 | 6.79 | 9.00 |
| 2 | 7.99 | 6.53 | 7.73 | 10.13 |
| 3 | 8.80 | 7.50 | 7.28 | 6.74 |
| 4 | 9.17 | 12.08 | 8.82 | 4.96 |
| 5 | 14.50 | 13.71 | 9.20 | 14.05 |
| 6 | 6.73 | 10.39 | 6.41 | 9.12 |
| 7 | 4.78 | 8.00 | 4.10 | 13.83 |
| 8 | 6.03 | 6.41 | 10.57 | 11.76 |
| 9 | 6.42 | 10.05 | 6.90 | 9.73 |
| 10 | 8.21 | 14.47 | 6.90 | 4.98 |
| 11 | 10.98 | 7.67 | 8.46 | 7.46 |
| 12 | 5.08 | 8.26 | 9.55 | 5.92 |
| 13 | 10.45 | 6.77 | 6.06 | 6.36 |
| 14 | 7.10 | 5.35 | 6.20 | 9.49 |
| 15 | 8.83 | 6.48 | 9.04 | 8.60 |
| 16 | 4.12 | 4.09 | 11.89 | 5.64 |
| 17 | 7.29 | 7.28 | 8.71 | 7.31 |
| 18 | 7.27 | 6.52 | 10.48 | 11.78 |
| 19 | 6.55 | 10.54 | 6.87 | 8.69 |
| 20 | 10.36 | 7.41 | 6.40 | 5.72 |
| 21 | 8.85 | 8.73 | 14.43 | 5.15 |
| 22 | 6.35 | 5.70 | 7.10 | 6.13 |
| 23 | 4.37 | 6.51 | 6.86 | 6.03 |
| 24 | 3.89 | 5.11 | 10.98 | 15.05 |
| 25 | 6.86 | 8.71 | 9.61 | 13.60 |
| 26 | 9.03 | 9.60 | 12.38 | 8.91 |
| 27 | 8.30 | 12.72 | 11.26 | 11.20 |
| 28 | 8.39 | 6.99 | 11.00 | 13.71 |
| 29 | 8.81 | 5.66 | 11.13 | 13.22 |
| 30 | 11.92 | 6.82 | 6.04 | － |
| 31 | 9.46 | 6.99 | 12.17 | － |
| 32 | 8.60 | 6.98 | 12.16 | － |
| 33 | － | － | 8.65 | － |
